# Synergistic interaction between cisplatin and taxol in human ovarian carcinoma cells in vitro.

**DOI:** 10.1038/bjc.1994.55

**Published:** 1994-02

**Authors:** A. P. Jekunen, R. D. Christen, D. R. Shalinsky, S. B. Howell

**Affiliations:** Department of Radiotherapy and Oncology, Helsinki University Central Hospital, Finland.

## Abstract

**Images:**


Br. J. Cancer (1994), 69, 299–306 © Macmillan Press Ltd., 1994

### Synergistic interaction between cisplatin and taxol in human ovarian carcinoma cells *in vitro*

A.P. Jekunen, R.D. Christen, D.R. Shalinsky & S.B. Howell

*Department of Radiotherapy and Oncology, Helsinki University Central Hospital, Helsinki, SF 00270, Finland; Department of Medicine, UCSD Cancer Center, University of California, San Diego, La Jolla, California 92 093-0812, USA.*

**Summary** Taxol, a unique tubulin active agent, was found to demonstrate a marked schedule-dependent synergistic interaction with cisplatin (DDP) in the killing of human ovarian carcinoma 2008 cells *in vitro* as determined by median effect analysis. The interaction was highly synergistic when 19 h taxol exposure was followed by 1 h concurrent exposure to taxol and DDP. The combination indices (CIs) on this schedule were 0.11 ± 0.1, 0.25 ± 0.15 and 0.39 ± 0.14 at 20%, 50% and 80% cell kill respectively. However, the interaction was antagonistic when 1 h exposure to DDP was followed by 20 h exposure to taxol, or when cells were exposed to DDP and taxol for 1 h concurrently. When taxol preceded DDP, synergy was also observed with the 11-fold DDP-resistant 2008/C13*5.25 subline, which yielded CI values of 0.21 ± 0.02, 0.30 ± 0.11 and 0.31 ± 0.17 at 20%, 50% and 80% cell kill respectively. At an $IC_{50}$ concentration, taxol had no effect on [$^3$H]*cis*-dichloro(ethylenediamine) platinum uptake, on the permeability of the plasma membrane or on glutathione or metallothionein levels in 2008 or 2008/C13*5.25 cells. Mitotic arrest in these cells was observed only at taxol concentrations well above those required for synergy with DDP, suggesting that the mechanism underlying the synergistic interaction was not a taxol-induced alteration in cell cycle kinetics. Of additional interest was the fact that the 2008/C13*5.25 cells were hypersensitive to taxol, and that this was partially explained by an alteration in the biochemical pharmacology of taxol. Although cellular taxol accumulation reached steady state within 2 h in both cell lines, taxol efflux was slower and the taxol was more extensively bound in 2008/C13*5.25 cells than in 2008 cells. In addition, the 2008/C13*5.25 cells had only 55% of the parental levels of β-tubulin content. However, in another pair of DDP-sensitive and -resistant ovarian cell lines no taxol hypersensitivity and no change in β-tubulin content was observed, indicating that the DDP-resistant and taxol-hypersensitive phenotypes do not segregate together. We conclude that taxol interacts synergistically with DDP in a manner that is highly schedule dependent, and that the hypersensitivity of 2008/C13*5.25 cells to taxol is unrelated to the mechanism of synergy. These *in vitro* observations suggest that drug schedule will be an important determinant of the activity and toxicity of the DDP and taxol drug combination in clinical studies.

DDP[3] often produces good initial therapeutic responses in ovarian carcinoma, but resistance to DDP develops frequently, leading to chemotherapeutic failure. The mechanisms reported to be capable of mediating DDP resistance include increased cellular glutathione, increased metallothionein, decreased drug uptake and Pt-DNA adduct formation, and enhanced DNA repair (Andrews & Howell, 1990). Because of the clinical importance of DDP, there has been wide interest in identifying agents that synergistically modulate DDP activity at the cellular level. Only a few such agents are known. Synergistic interactions have been reported for DDP with cytarabine (Andrews *et al.*, 1988; Keane *et al.*, 1990), 2′-deoxy-5-azacytidine (Frost *et al.*, 1990), and alkylating agents (Frost *et al.*, 1990; Lidor *et al.*, 1991; Hayward *et al.*, 1992). Anguidine (Hromas *et al.*, 1984) and forskolin (Mann *et al.*, 1991) have been noted to enhance the cellular uptake of DDP, whereas uptake is blocked by aldehydes (Dornish & Pettersen, 1985; Dornish *et al.*, 1986) and ouabain (Andrews *et al.*, 1988). Dipyridamole has been identified as another agent capable of enhancing sensitivity to DDP (Keane *et al.*, 1990; Jekunen *et al.*, 1992). Furthermore, quercetin, tamoxifen and staurosporin have been found to interact synergistically with DDP in Walker rat carcinoma cells (Hofmann *et al.*, 1989). The new phospholipid analogue BM41440 inhibits protein kinase C and is reported to demonstrate synergy with DDP (Hofmann *et al.*, 1989).

Anti-microtubule agents are among the most important anti-cancer drugs, and have significantly contributed to the therapy of most curable neoplasms, such as Hodgkin's and non-Hodgkin's lymphomas, germ cell tumours and childhood leukaemias. While other anti-microtubule agents induce microtubule disassembly, taxol promotes microtubule assembly. Taxol has demonstrated a broad anti-tumour spectrum in preclinical studies (McQuire *et al.*, 1989; Rowinsky *et al.*, 1990), and is active against DDP-unresponsive advanced ovarian cancer, with a reported response rate of 24–33% in phase II studies (Einzig *et al.*, 1990).

Several studies of the combination of DDP and taxol for the treatment of ovarian carcinoma are currently under way. The goal of this study was to investigate the nature of the interaction between DDP and taxol, and to determine whether the interaction demonstrated any schedule dependency. We report here that there is a highly synergistic interaction between these two drugs, but that the synergy is also highly schedule dependent.

### Materials and methods

### *Cell lines*

Experiments were conducted using the human ovarian carcinoma line 2008 (DiSaia *et al.*, 1972) and an 11-fold DDP-resistant (2008/C13*5.25) subline generated by monthly incubation in DDP (Andrews *et al.*, 1985). The human ovarian carcinoma A2780 and A2780$^{cp}$ cell lines were supplied by R. Ozols (Fox Chase Cancer Institute, Philadelphia, PA, USA). Cells were maintained in RPMI-1640 supplemented with 5% fetal bovine serum and 2 mM L-glutamine without antibiotics. Cultures routinely tested negative for mycoplasma by using Gen Probe kit (Gen Probe, San Diego, CA, USA).

### *Clonogenic assays*

Cells growing in log phase were harvested by trypsinisation, washed, dispersed and plated onto 60 mm plastic tissue culture dishes (Corning Glass Works, New York, NY, USA) in

Correspondence: S.B. Howell, UCSD Cancer Center, Department of Medicine 0812, 9500 Gilman Drive, University of California, San Diego, La Jolla, CA 92093, USA.
Received 29 June 1993; and in revised form 15 October 1993.

triplicate at a density of 300 cells per dish. Cells were exposed to taxol for 19 h and then, while the taxol exposure continued, to DDP for an additional hour. Alternatively, after allowing cells to attach overnight, cultures were exposed to either taxol and DDP concurrently, or first to DDP for 1 h and then to taxol for 20 h. Cells were incubated in 5% carbon dioxide at 37°C for 10 days, and then fixed with methanol and stained using Giemsa dye in methanol. Clusters of more than 40 cells were counted as one colony; control dishes generally contained 100–130 colonies.

*Median effect analysis*

The nature of the interaction between DDP and taxol was assessed by median effect analysis (Chou & Talalay, 1986; Berenbaum, 1989). Dose–response curves were determined for each agent alone, and with both agents in combination at a fixed ratio equivalent to the ratio of their $IC_{50}$ values. Computer analysis of the dose–response curves was used to calculate the CI at the level of 50% cell kill. Values of $<1$ indicate synergy, a value of 1 indicates additivity and values $>1$ indicate antagonism. Each point in the figures presented represents the mean of three separate experiments using triplicate cultures (s.e.).

*Electron microscopy*

Monolayer cells were treated with 10 nM taxol for 24 h, then fixed first in 2.5% (v/v) glutaraldehyde in 0.1 M sodium phosphate buffer (pH 7.4, total osmolarity 0.52 osmol) and then in 1% osmium tetroxide. Sections were cut parallel to the culture surface on an LKB-V ultramicrotome (Pharmacia LKB Biotechnology, Piscataway, NJ, USA). Thin sections were stained with saturated uranyl acetate in 50% (v/v) ethanol and then with bismuth subnitrate (Riva, 1974). Cells were viewed with an H-600 transmission electron microscope (Hitachi Instruments, Danbury, CT, USA). Sections of control and taxol-treated cells taken parallel to the plane of the tissue culture dish were used to estimate the volume density of microtubule bundles by point counting (Weibel, 1979; Mathieu-Costello, 1987). Measurements were made at a final magnification of 16,200 by placing a transparent overlay of a 400 point grid on random micrographs of cell sections taken in the region of the nucleus. Four sections of four random micrographs were analysed for each condition.

*Intracellular pharmacology of taxol*

Taxol uptake was determined using [$^3$H]taxol. One million cells were seeded in 60 mm tissue culture dishes, and after 3 days, when cell cultures were in log phase growth, the influx and efflux of taxol was determined. The medium was aspirated, and fresh medium containing 0.1 $\mu$Ci ml$^{-1}$ (27.8 nM) taxol was added for appropriate times to determine influx. Cells were then washed three times with ice-cold PBS. One millilitre of 1 nM sodium hydroxide was added and the cells allowed to digest overnight. An aliquot was removed for the determination of protein content by the method of Bradford (1976) and 0.5 ml was used for liquid scintillation counting.

In experiments designed to quantitate efflux, cells were first loaded by incubating them in taxol (27.8 nM, 0.1 $\mu$Ci ml$^{-1}$) for 2 h, and then the medium was aspirated and fresh medium containing no taxol was added. The efflux of taxol was expressed as the percentage of taxol radioactivity remaining in the cells as a function of time. The fraction of [$^3$H]taxol bound in 2008 and 2008/C13*5.25 cells was determined by ultrafiltration. The cells were first scraped from the dish into PBS, and then sonicated at 4°C. One aliquot of lysate was digested overnight in 1 ml of 1 M sodium hydroxide and then used for protein determination and for radioactivity measurement after neutralisation by 1 nM hydrocholoric acid. The remaining lysate was ultrafiltered for 30 min at 4°C using Amicon CF25 cones (Amicon, Beverley, MA, USA), and the radioactivity in the ultrafiltrate was determined by liquid scintillation counting.

*Effect of taxol on cellular permeability*

The effect of taxol on plasma membrane permeability was tested by trypan blue exclusion. Log-phase cells were trypsinised and resuspended at 500,000 cells ml$^{-1}$ in trypan blue-containing fresh medium to which taxol had been added. Cell staining after treatment with 10 nM, 100 nM and 1 $\mu$M taxol was followed by microscopy over 5 min. Further experiments were done by adding trypan blue following a 20 h incubation with 100 nM and 1 $\mu$M taxol, and subsequently examining the cells for uptake of trypan blue.

*Effect of taxol on cell cycle phase distribution*

Cells in the log phase of growth were exposed to 0, 10, 100 nM and 2 $\mu$M taxol for 20 h, then harvested by trypsinisation, washed in PBS containing 2 mM magnesium chloride, and fixed by adding 10 ml of 70% ethanol. Thereafter, cells were kept at 4°C until flow cytometric analysis. Cells were resuspended in 50 $\mu$M propidium iodide and 1,000 units ml$^{-1}$ RNAse A in PBS, incubated at 37°C for 30 min, and analysed on a Cytofluorograf (Ortho Diagnostics Systems, Raritan, NJ. USA). Multicycle Cell Cycle Software (Phoenix Flow Systems, San Diego, CA, USA) was used to calculate the fraction of cells in each phase of the cell cycle. This programme is based on the mathematical model described by Dean and Jett (1974), using Gaussian distribution functions for $G_1$ and $G_2$/M phases, and a broadened second-degree polynomial for S-phase. To assess the percentage of cells in M-phase, a method based on difference in chromatin structure was used (Darzynkiewicz *et al.*, 1977*a*,*b*). After fixation and treatment with RNAse, cells were treated with acid and stained with the metachromatic fluorochrome acridine orange (Larsen *et al.*, 1986). The extent of DNA denaturation per cell was estimated from the ratio of red to green fluorescence intensity. Metaphase and interphase cells were discriminated on the basis of their red/green intensity ratios after partial DNA denaturation.

*Determination of GSH content*

Log-phase 2008 and 2008/C13*5.25 cells were treated with taxol for 20 h before being harvested by trypsinisation, washed with PBS, aliquoted in triplicate at 250,000 cells per Eppendorf tube and pelleted for 2 min at 2,000 r.p.m. The medium was removed and 25 $\mu$l of MCB–Tris (2 mM MCB, 20 mM Tris–HCl, pH 8.0) was added, mixed and allowed to react for 15 min in the dark. An equal volume (30 $\mu$l) of sodium methanesulphonate was then added to lyse the cells and to precipitate cellular proteins. The GSH-containing supernatant was assayed by the high-performance liquid chromatography (HPLC) method of Fahey (1989) using 25 $\mu$l injection volumes and a Waters $C_{18}$ $\mu$-Bondapak cartridge and precolumn. Data collection and processing were carried on a Waters Maxima 820 system. Run time was 35 min with a retention time of 10 min.

*Northern analysis of metallothionein mRNA*

Total cellular RNA was harvested and electrophoresed in formaldehyde gels, and blots containing 5 $\mu$g per lane were prepared on Nitroplus Membranes (Micron Separations, Wesborough, MA, USA) (Davis *et al.*, 1986; Sambrook *et al.*, 1989). Membranes were hybridised sequentially with $^{32}$P-labelled cDNA probes for human metallothionein II (Karin & Richards, 1982) and $\beta$-actin (Gunning *et al.*, 1983), the latter used to control for lane loading.

*Quantitation of tubulin subunits*

Monoclonal antibodies directed against $\alpha$- and $\beta$-tubulin were used to quantitate tubulin subunits. Cells were harvested by trypsinisation, fixed in 37% (v/v) formaldehyde in PBS (pH 7.4) for 10 min at room temperature and permeabilised with 0.1% saponin in PBS for 5 min. Cells were incubated with 100 nM monoclonal antibody in 3% bovine serum albumin in PBS. After washing, the cells were stained with a fluorescent goat anti-mouse IgG/IgM antibody for 45 min in the dark, and then analysed on a CytoFluorograf (Ortho Diagnostics Systems, Raritan, NJ, USA) with excitation and emission settings of 488 and 530 nM respectively.

### Results

### *Interaction between DDP and taxol*

Figure 1 shows the clonogenic survival curves for DDP alone, taxol alone, and the combination of DDP and taxol at a fixed molar ratio of 7,500:1. The more negative slope of the curve for the combination suggests an interaction between the two drugs, but the nature of the interaction could not readily be discerned since both agents were independently cytotoxic (Berenbaum, 1989).

The nature of the interaction between taxol and DDP was determined by using median effect analysis (Chou & Talalay, 1986), which provides a tool to examine the degree of synergy, additivity or antagonism at various levels of cell kill. Median effect analysis permits calculation of the CI at various levels of cell kill. CI values of 1 indicate additivity, values of <1 synergy and values of >1 antagonism; the smaller the value of the CI below 1 the greater the degree of synergy, and the greater the value above 1 the greater the antagonism. Figure 2 shows the CI plot for some three separate experiments as shown in Figure 1 in which DDP and taxol were combined in a ratio of 7,500:1, which was the ratio of their individual $IC_{50}$ values. The curve demonstrates synergy between DDP and taxol in the killing of 2008 cells under conditions in which the cells were first treated with taxol for 19 h and then with DDP and taxol concurrently for 1 h. At the 20% level of cell kill, the average CI was 0.11 ± 0.1 (s.e.m.; $n = 3$), indicating synergy. At the 50% level of cell kill the mean CI was 0.25 ± 0.15 (s.e.m.; $n = 3$), and it was 0.39 ± 0.14 (s.e.m.; $n = 3$) at the 80% level of cell kill. The CI was significantly less than 1 ($P<0.05$) at all three levels of cell kill, and these values indicate a very strongly synergistic interaction.

Figure 3 shows the CI plot for the DDP-resistant 2008/C13*5.25 cells also exposed to DDP and taxol in a ratio of 250:1 using the same schedule of pretreatment with taxol for 19 h followed by 1 h concurrent exposure to taxol and DDP. Interestingly, these DDP-resistant cells were 5-fold hypersensitive to taxol. The CI values for the 2008/C13*5.25 cells were 0.21 ± 0.02, 0.30 ± 0.11 and 0.31 ± 0.17 at 20%, 50% and 80% cell kill respectively. Thus, synergy was evident and was statistically significant ($P<0.05$) over a range of 1 log of cell kill in both cell lines, and hypersensitivity to taxol did not affect the nature of the interaction between DDP and taxol.

**Figure 1** Percentage survival as a function of drug concentration for 2008 cells exposed to DDP for 1 h (circles), taxol for 19 h (squares), and the combination of DDP and taxol, exposed first to taxol for 19 h and then to taxol and DDP for 1 h at a fixed molar ratio of 250:1 (triangles). Each point in all plots represents the mean of three experiments using triplicate cultures (s.e.).

**Figure 2** CI as a function of extent of cell kill for 2008 cells exposed first to taxol for 19 h and then to taxol and DDP for 1 h at a molar ratio of 1:250. Each point in all plots represents the mean of three separate experiments using triplicate cultures (s.e.).

**Figure 3** CI as a function of extent of cell kill for 2008/C13*5.25 cells exposed first to taxol for 19 h and then to DDP and taxol for 1 h at a molar ratio of 7,500:1.

Figure 4 presents the CI curve for 2008 cells when the same ratio of drugs was employed but on a schedule in which the cells were first exposed to DDP for 1 h, following which the medium was changed and the cells exposed to taxol for 20 h. This treatment schedule resulted in substantial antagonism instead of the synergy over the first 2 logs of cell kill.

Figure 5 shows the CI plot resulting from experiments in which 2008 cells were exposed concurrently to DDP and taxol for only 1 h. On this schedule, the $IC_{50}$ concentrations for DDP and taxol were 2.5 μM and 50 nM, and because of limits on the solubility of taxol the experiments were performed at a DDP/taxol concentration ratio of 50:1. The CI plot indicates antagonism between the two drugs on this schedule.

### *Effect of taxol on glutathione levels*

The effect of a 20 h pretreatment with an $IC_{50}$ concentration of taxol on the GSH content of 2008 and 2008/C13*5.25 cells is shown in Table I. The GSH levels were 2-fold higher in the DDP-resistant cells ($P = 0.00098$, two-tailed unpaired $t$-test), but taxol pretreatment did not alter the glutathione level in either line.

*Effect of taxol on cell cycle phase distribution*

Sensitivity to DDP varies in different phases of the cell cycle, being greatest in $G_1$ (Roberts & Fravel, 1980). One mechanism mediating the schedule dependency of the synergistic interaction between DDP and taxol may be taxol-induced perturbation of the cell cycle phase distribution. In subconfluent 2008 cell cultures, <6% of the cells were in M-phase, approximately 40% were in S-phase, and the same percentage were in $G_1$ (Table II). Exposure of 2008 cells for 20 h to an $IC_{50}$ concentration of taxol caused no change in cell cycle phase distribution when assessed at time points out to 72 h (data not shown). Likewise, the distribution did not change when cells were exposed for 20 h to a concentration 10 times the $IC_{50}$. In contrast, exposure of the cells to a taxol concentration of 2 μM (200 times the $IC_{50}$) for 20 h blocked approximately 50% of the cells in M-phase. Twenty-four hours after the end of exposure to 2 μM taxol (i.e. at hour 44) some additional $G_2$ arrest was apparent as 86.5% of the cells were in $G_2$/M-phase, but only 25% were in M-phase. Thus at concentrations at which synergy with DDP was apparent, taxol did not have any measurable effect on the cell cycle phase distribution.

**Figure 4** CI as a function of extent of cell kill for 2008 cells exposed first to DDP and then taxol for 20 h at a molar ratio of 1:250.

**Figure 5** CI as a function of extent of cell kill for 2008 cells exposed concurrently to DDP and taxol for 1 h at a DDP/taxol molar ratio of 1:50.

**Table I** Effect of taxol on GSH content

| *Cell type* | *Taxol* | *GSH content*[a] |
|---|---|---|
| 2008 | – | 32.6 ± 6.0 |
| | + | 34.5 ± 4.7 |
| 2008/C13*5.25 | – | 63.7 ± 23.2 |
| | + | 53.8 ± 18.5 |

[a]μmol per 2.5 × $10^5$ cells.

*Effect of taxol on microtuluble polymerisation*

Despite the fact that very high concentrations of taxol were required to produce M-phase arrest, Figure 6 shows that a 24 h exposure to even 10 nM taxol (equivalent to the $IC_{50}$) was sufficient to cause characteristic formation of microtubule bundles in 2008 cells. The volume density of bundles was determined by stereological analysis of electron micrographs. Cells attached to plastic dishes were cut parallel to the plane of the dish in a region including the nucleus. The volume density of microtubule bundles was 5 ± 3% and 11 ± 5% in the control and taxol-treated 2008 cells respectively ($P < 0.04$, two-sided $t$-test). Thus, at concentrations at which taxol demonstrated synergy with DDP, it was capable of altering microtubular morphology even though not capable of causing cell cycle arrest.

*Taxol effects on metallothionein levels measured by Northern analysis*

A 20 h treatment with an $IC_{50}$ taxol concentration had no effect on metallothionein $II_A$ message levels in 2008 or 2008/C13*5.25 (data not shown). A 50-fold DDP-resistant subline, 2008/C13*50, which constitutively overexpresses metallothionein $II_A$, was included as a positive control. A 20 h exposure to an $IC_{50}$ concentration of taxol did not increase metallothionein $II_A$ expression in this line either.

*Hypersensitivity of DDP-resistant cells to taxol*

Separate from the fact that DDP and taxol exhibit schedule-dependent synergy, the observation that the DDP-resistant 2008/C13*5.25 cells were actually 5-fold hypersensitive to taxol is of substantial interest with regard to the use of these drugs in combination. As a first step towards identifying the mechanism underlying this collateral hypersensitivity we compared the two cell lines with respect to the biochemical pharmacology of taxol. Figure 7a shows the time course of taxol accumulation in 2008 and 2008/C13*5.25 cells; consistent with our previous results (Christen *et al.*, 1993), there was no difference in either the initial influx of [$^3$H]taxol or steady-state content. Figure 5b shows the time course of efflux after the cells had been loaded by incubation in taxol-containing medium for 120 min, then washed and allowed to efflux into taxol-free medium. At 30 min after the start of efflux, the 2008/C13*5.25 cells had lost 10% of their initial content of drug, whereas the 2008 cells had lost 53%. These results suggested that there was either a decreased efflux rate constant or increased intracellular binding of taxol in the 2008/C13*5.25 cells. Ultrafiltration experiments were done to determine the fraction of taxol-bound intracellularly at steady state. Table III shows that approximately 16% more drug was ultrafilterable in the 2008 than the 2008/C13*5.25 cells, both at steady state and at 90 min after the start of

**Table II** Effects of taxol on the cell cycle phase distribution of 2008 cells

| | *Control* | | *Taxol 10 nM* | *Taxol 100 nM* | | *Taxol 2,000 nM* | |
|---|---|---|---|---|---|---|---|
| | *20 h* | *44 h* | *20 h* | *20 h* | *44 h* | *20 h* | *44 h* |
| $G_1$ | 41.2 ± 3.0[a] | 41.0 ± 2.4 | 42.8 ± 1.2 | 42.3 ± 1.3 | 40.6 ± 1.6 | 11.3 ± 2.4 | 4.5 ± 1.3 |
| S | 43.9 ± 5.4 | 40.1 ± 2.4 | 44.1 ± 1.6 | 43.7 ± 2.4 | 41.6 ± 1.2 | 17.8 ± 2.6 | 9.0 ± 1.6 |
| $G_2$/M | 14.9 ± 3.6 | 18.9 ± 1.0 | 13.1 ± 2.8 | 14.0 ± 3.3 | 17.8 ± 0.7 | 70.9 ± 5.0 | 86.5 ± 1.2 |
| M | 1.6 ± 0.3 | 5.9 ± 1.5 | 1.4 ± 0.4 | 1.5 ± 0.3 | 4.8 ± 1.3 | 54.9 ± 4.5 | 23.7 ± 1.8 |

[a]Each point shows the mean of three experiments (± s.d.).

Figure 6 Transmission electron micrographs of 2008 cells exposed for 24 h to 10 nM taxol. Arrows indicate characteristic hyperpolymerised tubulin bundles. **a**, 2008 cell at 16,000 magnification. **b**, 2008 cell at 150,000 magnification.

**Figure 7** **a**, Uptake and **b**, Efflux of [$^3$H]taxol into and from 2008 cells (open circles) and 2008/C13*5.25 cells (closed circles). Each point represents the mean of three experiments performed with triplicate cultures. Vertical bars represent s.d.

**Table III** Percentage [$^3$H]taxol ultrafiltrable at steady state and during efflux

| | Cell line | |
|---|---|---|
| *Exposure conditions* | *2008* | *2008/C13*5.25* |
| Two hours incubation with taxol | 69.1 ± 8.9[a] | 53.4 ± 4.5 |
| Four hours incubation with taxol | 69.6 ± 6.0 | 53.3 ± 5.5 |
| Two hours incubation with taxol followed by 90 min efflux into drug-free medium | 68.4 ± 7.1 | 58.0 ± 2.7 |

[a]Each value is the mean of two experiments performed with quadruplicate cultures.

drug efflux. Thus, increased intracellular binding of taxol contributed to the difference in the extent of taxol efflux observed between the two cell lines, but other mechanisms are likely to be involved as well since the magnitude of the difference in intracellular binding is substantially less than the difference in the extent of efflux at 90 min. It should be noted that neither of the cell types expresses the *mdr1* gene detectable by either Northern blotting, PCR amplification or immunohistochemical staining (data not shown). However, 2008/C13*5.25 cells do demonstrate an abnormality of tubulin content. They contain only 55 ± 8% (s.d.) of the parental content of β-tubulin ($P < 0.0002$, two-sided paired $t$-test), while α-tubulin content is unchanged (Christen *et al.*, 1993).

To determine whether hypersensitivity to taxol was a consistent feature of the DDP-resistant phenotype in ovarian carcinoma cell lines, we examined the taxol sensitivity of another pair of DDP-sensitive and -resistant human ovarian carcinoma cells, A2780 and A2780$^{cp}$ cells. While the A2780$^{cp}$ cells are 6-fold resistant to DDP, they showed no difference in sensitivity to taxol using a 20 h exposure (Figure 8). In addition, they showed no abnormality of tubulin content. The A2780$^{cp}$ cells contained 92 ± 17% and 95 ± 13%, respectively, of the α- and β-tubulin present in the parental 2780 cells. Thus, taxol hypersensitivity does not co-segregate with the DDP-resistant phenotype in ovarian carcinoma cell lines.

### Discussion

These studies demonstrated a highly synergistic cytotoxic interaction between DDP and taxol, but also demonstrated that this interaction was very schedule dependent. Synergy was noted only when taxol exposure preceded a 1 h exposure to DDP. Both a 1 h concurrent exposure to both drugs and a 1 h exposure to DDP followed by a 20 h exposure to taxol produced an antagonistic interaction. These results have important clinical implications for the use of these two drugs in combination for the treatment of ovarian carcinoma. First, they suggest that the optimal anti-tumour effect will be

**Figure 8** Clonogenic survival as a function of concentration for A2780 (open circles) and A2780[cp] (closed circles) cells following **a**, a 1 h exposure to DDP or **b**, a 20 h exposure to taxol. Each point represents the mean of three experiments, each conducted with triplicate cultures.

obtained through a schedule which permits some time for establishment of taxol effect before the administration of DDP. Second, since synergy was observed with both DDP-sensitive and DDP-resistant cells, it may be possible to take advantage of the synergistic interaction in both newly diagnosed patients with DDP-sensitive tumours as well as patients failing primary chemotherapy with DDP or its analogues. It should be emphasised that taxol and DDP may interact synergistically with respect to toxicity to normal tissues as well, and this may necessitate clinical use of a less than fully synergistic schedule.

Administration of taxol before DDP was also shown to result in greater anti-tumour activity than the reverse schedule when tested against L1210 murine leukaemia cells *in vitro* by Citardi *et al.* (1990). In these cells, a 24 h exposure to taxol followed by a 30 min exposure to DDP produced greater cell kill than either concurrent exposure to both drugs or a sequence consisting of a 30 min exposure to DDP followed by a 24 h exposure to taxol. The relative DDP $LD_{90}$ values in the presence of taxol were 28%, 100% and 83% of those for DDP alone on the three schedules. Rowinsky *et al.* (1991) showed in a phase I trial that myelosuppression was less profound when taxol was given prior to DDP compared with the reverse schedule. However, this difference in toxicity was associated with a higher clearance of taxol when it was given before rather than after DDP, and the effect of one drug on the sensitivity to the other at the cellular level could not be assessed. Nevertheless, based on these data the sequence of taxol administered prior to DDP was selected for further clinical development (Rowinsky & Donehower, 1991).

DDP sensitivity is related to the extent of drug uptake, and in fact in studies reported elsewhere (Christen *et al.*, 1993) we have shown that a 20 h exposure to taxol is capable of causing a concentration-dependent increase in the uptake of [$^3$H]DEP, a tritiated analogue of DDP. This effect was produced in the absence of any plasma membrane damage as measured by the ability of the cells to exclude trypan blue, indicating that the increase in [$^3$H]DEP uptake was not due to a non-specific increase membrane permeability. However, the concentrations of taxol required to produce increased uptake (100 nM to 1 μM) were >10-fold above the $IC_{50}$. Taxol was able to produce synergistic killing of cells with DDP at concentrations that were far below those required to increase [$^3$H]DEP uptake.

Although cellular content of GSH has been reported to be one determinant of DDP sensitivity, taxol caused no change in the GSH level of either 2008 or 2008/C13*5.25 cells. Likewise, concentrations of taxol capable of interacting synergistically caused no increase in metallothionein messenger RNA, and by inference total metallothionein content.

Taxol characteristically causes cells to arrest in M-phase. One might hypothesise that taxol was interacting synergistically with DDP by virtue of its ability to cause cells to accumulate in a particularly DDP-sensitive portion of the cell cycle. However, cells have been reported to be most sensitive to DDP in the $G_1$ phase of the cell cycle (Drewinko *et al.*, 1980), and taxol caused no detectable perturbation of the cell cycle phase distribution of 2008 cells at the concentrations required for synergy.

Although we have been unable to identify the mechanism underlying the schedule-dependent synergistic interaction between DDP and taxol, it is noteworthy that both drugs have been reported to interact with tubulin. Taxol characteristically causes a stabilisation of tubulin, resulting in hyperpolymerisation (Schiff *et al.*, 1979), and in fact does so in 2008 cells even at concentrations as low as 10 nM. DDP has been reported by Rixe *et al.* (1993) to cause polymerisation of tubulin, but by Peyrot *et al.* (1986) to cause depolymerisation. Further evidence for an effect of DDP on tubulin comes from the fact that 2008/C13*5.25 cells have both a decrease in β-tubulin and altered tubulin structure visible on confocal microscopy (Christen *et al.*, 1993). We speculate that the synergistic interaction may be occurring at the protein rather than at the DNA level.

It is equally of interest that 1 h DDP exposure was able to decrease the sensitivity of cells to a subsequent exposure to taxol, resulting in strong antagonism between the two drugs with respect to cytotoxicity even at low concentrations of taxol. Given the fact that DDP-induced $G_2$ arrest does not become manifest for >24 h in 2008 cells, and that it requires quite high concentrations of taxol to produce the characteristic M-phase arrest, it is unlikely that the antagonism was on the basis of cytokinetic perturbations, but one can speculate that DDP might be altering the tubulin binding site for taxol or that taxol-induced alterations in tubulin could be interfering with DDP DNA formation or persistence. Taxol concentrations as low as 1 nM have been shown to promote tubulin polymerisation and shift the equilibrium in favour of microtubule assembly (Roytta *et al.*, 1987).

Unexpectedly, the 2008/C13*5.25 cells were found to be 5-fold hypersensitive to taxol. Biochemical pharmacology studies showed that this hypersensitivity was associated with a markedly reduced efflux of taxol following drug loading, a small increase in intracellular binding and the above-mentioned changes in β-tubulin content and structure (Christen *et al.*, 1993). However, study of the A2780 and A2780[cp] cell lines demonstrated that this taxol-hypersensitive phenotype did not segregate with that of DDP resistance, and thus we conclude that the taxol hypersensitivity of the 2008/C13*5.25 cells was due to an abnormality of tubulin unique to these particular cells. DDP is mutagenic (Chibber & Ord, 1989), and it is conceivable that during the process of selection for resistance to DDP 2008/C13*5.25 cells acquired alterations in one or more tubulin or tubulin-associated protein genes. In several yeast models it has been shown that the accumulation of α-tubulin in excess over β-tubulin is uniquely toxic because it interferes with normal microtubule assembly

(Weinstein & Solomon, 1990). Overexpression of β-tubulin resulted in loss of normal microtubular structures and viability, but an excess of α-tubulin over β-tubulin was not toxic (Weinstein & Solomon, 1990). Given that the ratio of α- to β-tubulin was no different in the A2780$^{cp}$ than in the A2780 cells, whereas the amount of β-tubulin in 2008/C13*5.25 cells was decreased relative to that of α-tubulin (Christen *et al.*, 1993), it is conceivable that the response to taxol was also altered.

Peak plasma concentrations of taxol range from a mean of 0.95 μM for 275 mg m$^{-2}$ given as a constant rate infusion over 24 h to >8 μM when given at a dose of 275 mg m$^{-2}$ over 6 h (Wiernik *et al.*, 1987*a,b*). Thus, the attainable plasma concentrations of taxol are well above the range necessary to demonstrate synergistic killing when combined with DDP. The schedule-dependent synergy between these two drugs will further complicate the clinical assessment of combination taxol/DDP therapy for patients with ovarian cancer.

The authors would like to thank Susan Horowitz PhD for providing [$^3$H]taxol, Mr Dennis Heath for assistance with GSH measurements and cell cycle determinations and Mr Dennis Young for assistance with flow cytometry experiments.

This work was supported in part by the Academy of Finland, Grant CA 08993 from the National Institutes of Health, Grant CH377 from the American Cancer Society, grants from the Swiss National Science Foundation and the Swiss Cancer League and Grant 100R40 from Bristol-Myers Squibb Inc. This work was conducted in part by the Clayton Foundation for Research – California Division. Drs Jekunen, Christen and Howell are Clayton Foundation investigators.

**Abbreviations:** CI, combination index; $CI_{50}$, combination index at 50% cell kill; DDP, cisplatin; DEP, dichloro(ethylenediamine)-platinum(II); GSH, glutathione; MCB, monochlorobromobimane; PBS, phosphate-buffered saline pH 7.8; Pt, platinum; $^3$H, tritium.

### References

ANDREWS, P.A. & HOWELL, S.B. (1990). Cellular pharmacology of cisplatin: perspectives on mechanisms of acquired resistance. *Cancer Cells*, **2**, 93–100.

ANDREWS, P.A., MURPHY, M.P. & HOWELL, S.B. (1985). Differential potentiation of alkylating and platinating agent cytotoxicity in human ovarian carcinoma cells by glutathione depletion. *Cancer Res.*, **45**, 6250–6253.

ANDREWS, P.A., VELURY, S., MANN, S.C. & HOWELL, S.B. (1988). *cis*-diamminedichloroplatinum(II) accumulation in sensitive and resistant human ovarian carcinoma cells. *Cancer Res.*, **48**, 68–73.

BERENBAUM, M.C. (1989). What is synergy? *Pharmacol. Rev.*, **41**, 93–142.

BRADFORD, M.M. (1976). A rapid and sensitive method for the quantitation of microgram quantities of protein using the principle of protein-dye binding. *Anal. Biochem.*, **72**, 248–254.

CHIBBER, R. & ORD, M.J. (1989). The mutagenic and carcinogenic properties of three second generation antitumor platinum compounds: a comparison with cisplatin. *Eur. J. Cancer Clin. Oncol.*, **25**, 27–33.

CHOU, T.C. & TALALAY, P. (1986). Quantitative analysis of dose–effect relationships: the combined effects of multiple drugs or enzyme inhibitors. *Adv. Enzyme Reg.*, **22**, 27–55.

CHRISTEN, R.D., JEKUNEN, A.P., JONES, J.A., THIEBAUT, F., SHALINSKY, D.R. & HOWELL, S.B. (1993). *In vitro* modulation of cisplatin accumulation in human ovarian carcinoma cells by pharmacologic alteration of microtubules. *J. Clin. Invest.*, **92**, 431–440.

CITARDI, M.J., ROWINSKY, E.K., SCHAEFER, K.L. & DONEHOWER, R.C. (1990). Sequence-dependent cytotoxicity between cisplatin (C) and the antimicrotubule agents taxol (T) and vincristine (V) (abstract). *Proc. Am. Assoc. Cancer Res.*, **31**, 410.

DARZYNKIEWICZ, Z., TRAGANOS, F., SHARPLESS, T. & MELAMED, M.R. (1977*a*). Recognition of cells in mitosis by flow cytometry. *J. Histochem. Cytochem.*, **58**, 875–880.

DARZYNKIEWICZ, Z., TRAGANOS, F., SHARPLESS, T. & MELAMED, M.R. (1977*b*). Interphase and metaphase chromatin: different stainability of DNA with acridine orange after treatment at low pH. *Exp. Cell. Res.*, **110**, 201–214.

DAVIS, L.G., DIBNER, M.D. & BATTEY, J.F. (1986). Agarose gel electrophoresis. In *Basic Methods in Molecular Biology*, pp. 58–61. Elsevier: New York.

DEAN, P.N. & JETT, J.H. (1974). Mathematical analysis of DNA distributions derived from flow microfluorometry. *J. Cell Biol.*, **60**, 523–527.

DISAIA, P.J., SINKOVICS, F.N., RUTLEDGE, F.N. & SMITH, J.P. (1972). Cell-mediated immunity to human malignant cells. *Am. J. Obstet. Gynecol.*, **114**, 979–989.

DORNISH, J.M. & PETTERSEN, E.O. (1985). Protection from cis-dichloro-diammineplatinum-induced cell inactivation by aldehydes involves cell membrane amino groups. *Cancer Lett.*, **29**, 238–243.

DORNISH, J.M., MELVIK, J.E. & PETTERSEN, E.O. (1986). Reduced cellular uptake of cis-dichlorodiammine-platinum by benzaldehyde. *Anticancer Res.*, **6**, 583–588.

DREWINKO, B., CORRY, P., BERGERAT, J.-P. & BARLOGIE, B. (1980). Cells in different stages of the cell cycle. In *Cisplatin: Current Status and New Developments*, Prestayko, A.W. & Crooke, S.T. (eds), pp. 37–56. Academic Press: New York.

EINZIG, A.I., WIERNIK, P.H., SASLOFF, J., GARL, S., RUNOWICZ, C., O'HANLAN, K.A. & GOLDBERG, G. (1990). Phase II study of taxol in patients with advanced ovarian cancer. *Proc. Am. Assoc. Cancer Res.*, **31**, 187.

FAHEY, R.C. (1989). HPLC analysis of thiols using monobromobimane fluorescent labeling: application to radioprotective drugs. In *CRC Handbook of Free Radicals and Antioxidants in Biomedicine*, Vol. III, Miquel, J. (ed.), pp. 265–270. CRC Press: Boca Raton, FL.

FROST, P., ABBRUZZESE, J.L., HUNT, B., LEE, D. & ELLIS, M. (1990). Synergistic cytotoxicity using 2′-deoxy-5-azacytidine and cisplatin or 4-hydroperoxycyclophosphamide with human tumor cells. *Cancer Res.*, **50**, 4572–4577.

GUNNING, P., PONTE, P., OKAYAMA, H., ENGEL, J., BLAU, H. & KEDES, L. (1983). Isolation and characterization of full length cDNA clones for human alpha-, beta, and delta-action mRNAs: skeletal but not cytoplasmic actins have an amino-terminal cysteine that is subsequently removed. *Mol. Cell. Biol.*, **3**, 787–795.

HAYWARD, I.P., HURST, T., PARSONS, P.G. & KHOO, K.S. (1992). Combination chemotherapy tested in a short-term thymidine incorporation assay in primary cultures of ovarian adenocarcinomas. *Int. J. Cloning*, **10**, 182–189.

HOFMANN, J., UEBERALL, F., POSCH, L., MALY, K., HERRMANN, D.B. & GRUNICKE, H. (1989). Synergistic enhancement of the antiproliferative activity of cis-diamminedichloroplatinum(II) by the ether lipid analogue BM41440, an inhibitor of protein kinase C. *Lipids*, **24**, 312–327.

HROMAS, R., MEYN, R., JENKINS, S. & BARLOGIS, B. (1984). Anquidine enhances cis-platinum-induced DNA crosslinks in Chinese hamster ovary cells (abstract). *Proc. Am. Assoc. Cancer Res.*, **25**, 370.

JEKUNEN, A., VICK, J. SANGA, R., CHAN, T.C.K. & HOWELL, S.B. (1992). Synergism between dipyridamole and cisplatin in human ovarian carcinoma cells *in vitro*. *Cancer Res.*, **52**, 3566–3571.

KARIN, M. & RICHARDS, R.I. (1982). Human metallothionein genes – primary structure of the metallothionein-II gene and a related processed gene. *Nature*, **299**, 797–802.

KEANE, T.E., ROSNER, G., DONALDSON, J.T., NORWOOD, D.L., POULTON, S.H. & WALTHER, P.J. (1990). Dipyridamole–cisplatin potentiation: enhanced *in vivo* cytotoxicity in xenograft models of human testicular and bladder cancers. *J. Urol.*, **144**, 1004–1009.

LARSEN, J.K., MUNCH-PETERSEN, B., CHRISTIANSEN, J. & JORGENSEN, K. (1986). Flow cytometric discrimination of mitotic cells: resolution of M, as well as $G_1$, S, and $G_2$ phase nuclei with mithramycin, propidium iodide, and ethidium bromide after fixation with formaldehyde. *Cytometry*, **7**, 54–63.

LIDOR, Y.J., SHPALL, E.J., PETERS, W.P. & BAST, Jr, R.C. (1991). Synergistic cytotoxicity of different alkylating agents for epithelial ovarian cancer. *Int. J. Cancer*, **49**, 704–710.

MANN, S.C., ANDREWS, P.A. & HOWELL, S.B. (1991). Modulation of cis-diamminedichloroplatinum(II) accumulation and sensitivity by forskolin and 3-isobutyl-1-methylxanthine in sensitive and resistant human ovarian carcinoma cells. *Int. J. Cancer*, **48**, 866–872.

MCQUIRE, W.P., ROWINSKY, E.K., ROSENHEIN, N.B., GRUMBINE, F.C., ETTINGER, D.S., ARMSTRONG, D.K. & DONEHOWER, R.C. (1989). Taxol: a unique antineoplastic agent with significant activity in advanced ovarian epithelial neoplasms. *Ann. Intern. Med.*, **111**, 273–279.

MATHIEU-COSTELLO, O. (1987). Stereology. In *Handbook of Bioengineering*, Skalak, R. & Chien, S. (eds), pp. 38.1–38.31. McGraw-Hill: New York.

PEYROT, B., BRIAND, C., MOMBURG, R. & SARI, J.C. (1986). *In vitro* mechanism study of microtubule assembly inhibition by cis-dichlorodiammine-platinum(II). *Biochem. Pharmacol.*, **35**, 371–375.

RIVA, A. (1974). A simple and rapid staining method for enhancing the contrast of tissue previously treated with uranyl acetate. *J. Microsc.*, **19**, 105–108.

RIXE, O., ALVAREZ, M. MICKLEY, L., LY, H., PARKER, R., TSOKOS, M., REED, E. & FOJO, T. (1993). Cisplatin (CP) resistance is multifactorial and includes changes in cytoskeletal distribution and dynamics. *Proc. Am. Assoc. Cancer Res.*, **34**, 406.

ROBERTS, J.J. & FRAVEL, H.N.A. (1980). Repair of cis-platinum (II) diammine dichloride-induced DNA damage and cell sensitivity. In *Cisplatin: Current Status and New Developments*, Prestayko, A.W., Crooke, S.T. & Carter, S.K. (eds), pp. 57–78. Academic Press: New York.

ROWINSKY, E.K. & DONEHOWER, R.C. (1991). Taxol: twenty years later, the story unfolds. *J. Natl Cancer Inst.*, **83**, 1778–1781.

ROWINSKY, E.K., CAZENAVE, L.A. & DONEHOWER, R.C. (1990). Taxol: a novel investigational antimicrotubule agent. *J. Natl Cancer Inst.*, **82**, 1247–1259.

ROWINSKY, E.K., GILBERT, M.R., MCGUIRE, W.P., NOE, D.A., GROCHOW, L.B., FORASTIERE, A.A., ETTINGER, D.S., LUBEJKO, B.G., CLARK, B. & SARTORIUS, S.E. (1991). Sequences of taxol and cisplatin: a phase I and pharmacologic study. *J. Clin. Oncol.*, **9**, 1692–1703.

ROYTTA, M., LAINE, K.-M. & HARKONEN, P. (1987). Morphological studies on the effect of taxol on cultured human prostatic cancer cells. *Prostate*, **11**, 95–106.

SAMBROOK, J.T., MANIATIS, T. & FRITSH, E.F. (1989). In *Molecular Cloning: A Laboratory Manual*, Ford, N., Nolan, C. & Ferguson, M. (eds), pp. 7.39–7.52. Cold Spring Harbor Laboratory Press: Cold Spring Harbor, NY.

SCHIFF, P.P., FANT, J. & HORWITZ, S.B. (1979). Promotion of microtubule assembly *in vitro* by taxol. *Nature*, **22**, 665–667.

WEIBEL, E.R. (1979). *Sterological Methods, Practical Methods for Biological Morphometry*, Vol. I. Academic Press: London.

WEINSTEIN, B. & SOLOMON, F. (1990). Phenotypic consequences of tubulin overproduction in Saccharomyces cerevisiae: differences between alpha and beta tubulin. *Mol. Cell. Biol.*, **10**, 5295–5304.

WIERNIK, P.H., SCHWARTZ, E.L., EINZIG, A., STRAUMAN, J.J., LIPTON, R.B. & DUTCHER, J.P. (1987*a*). Phase I trial of taxol given as a 24 h infusion every 21 days: responses observed in metastatic melanoma. *J. Clin. Oncol.*, **5**, 1232–1239.

WIERNIK, P.H., SCHWARTZ, E.L., STRAUMAN, J.J., DUTCHER, J.P., LIPTON, R.B. & PAIETTA, E. (1987*b*). Phase I clinical and pharmacokinetics study of taxol. *Cancer Res.*, **47**, 2486–2493.


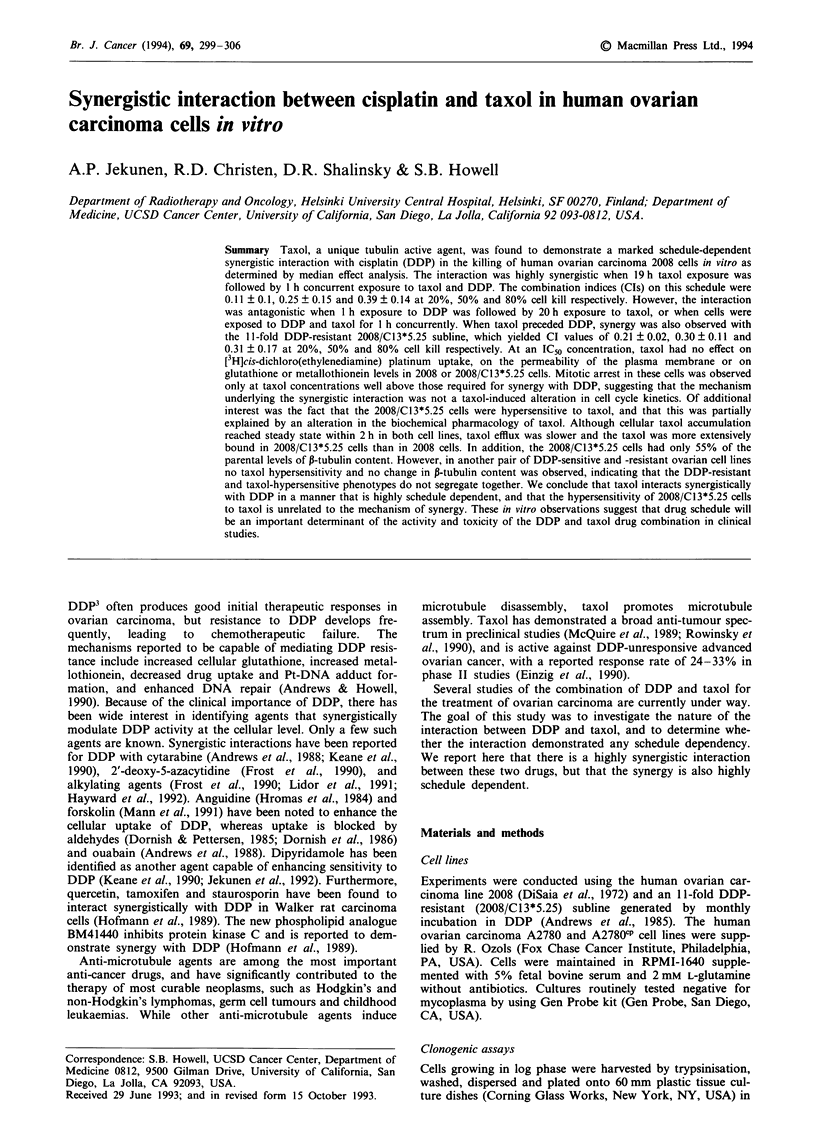

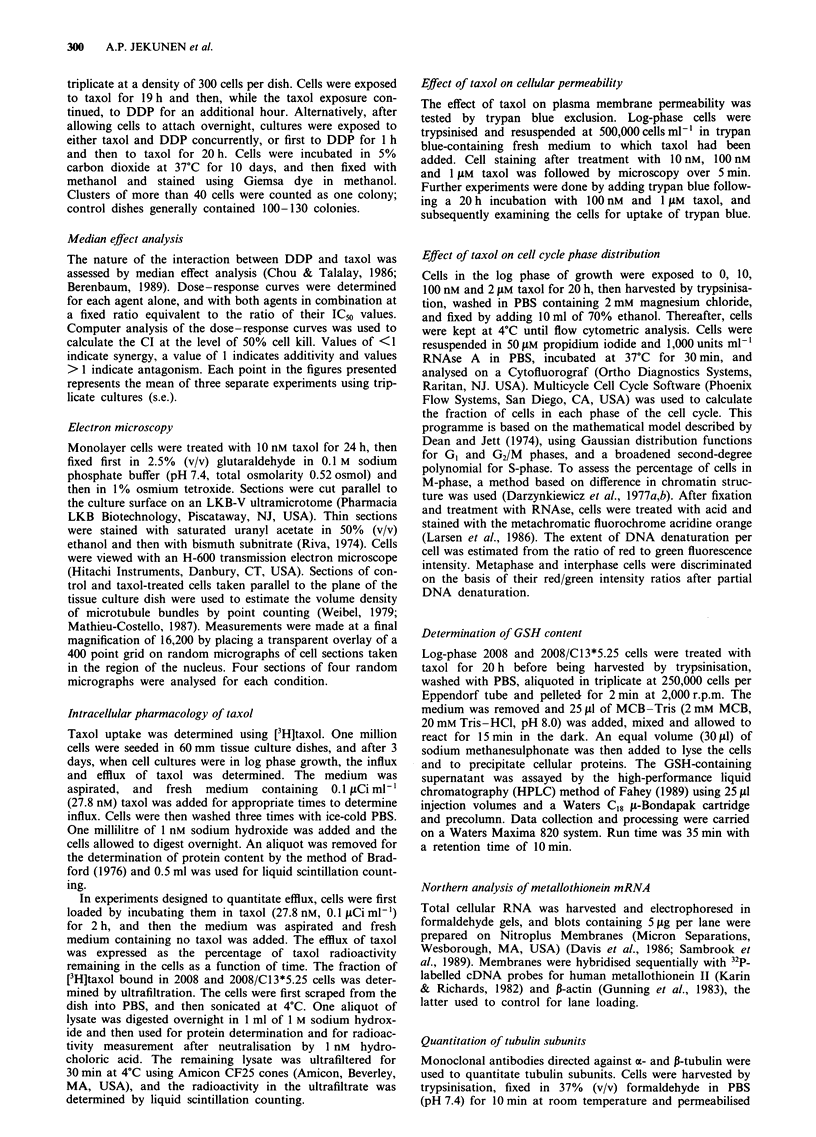

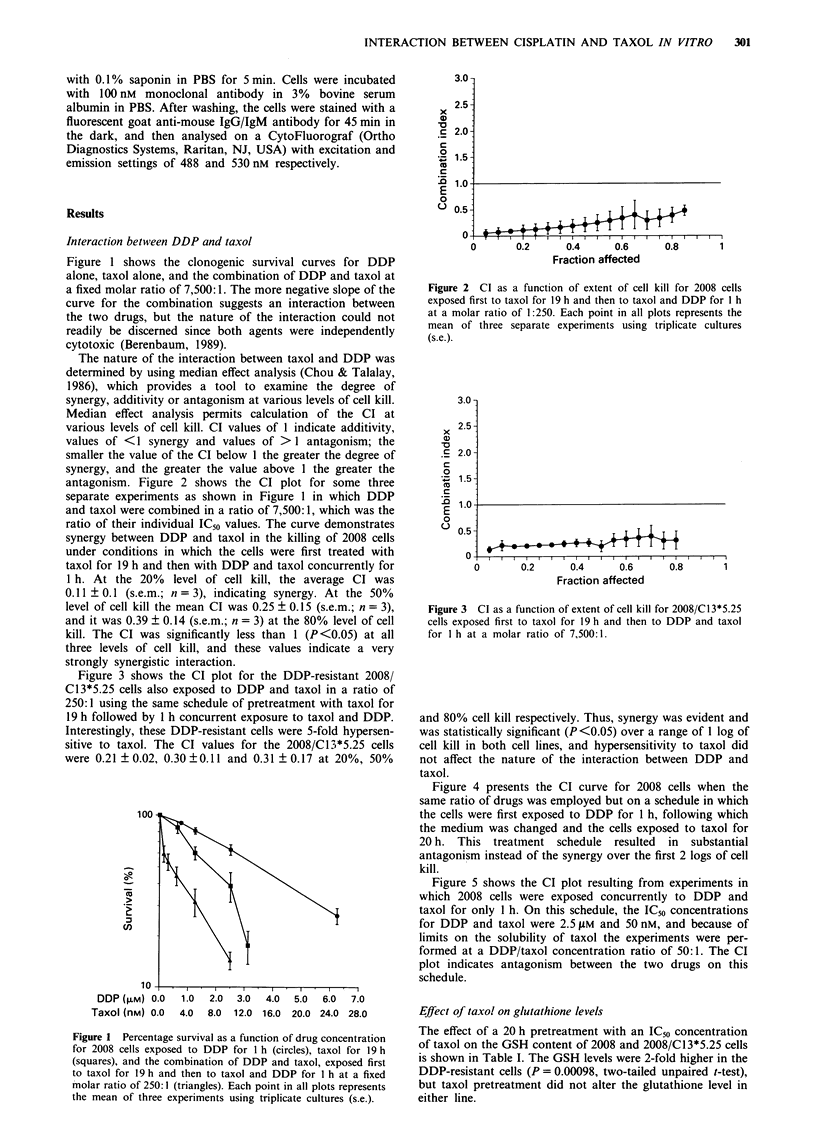

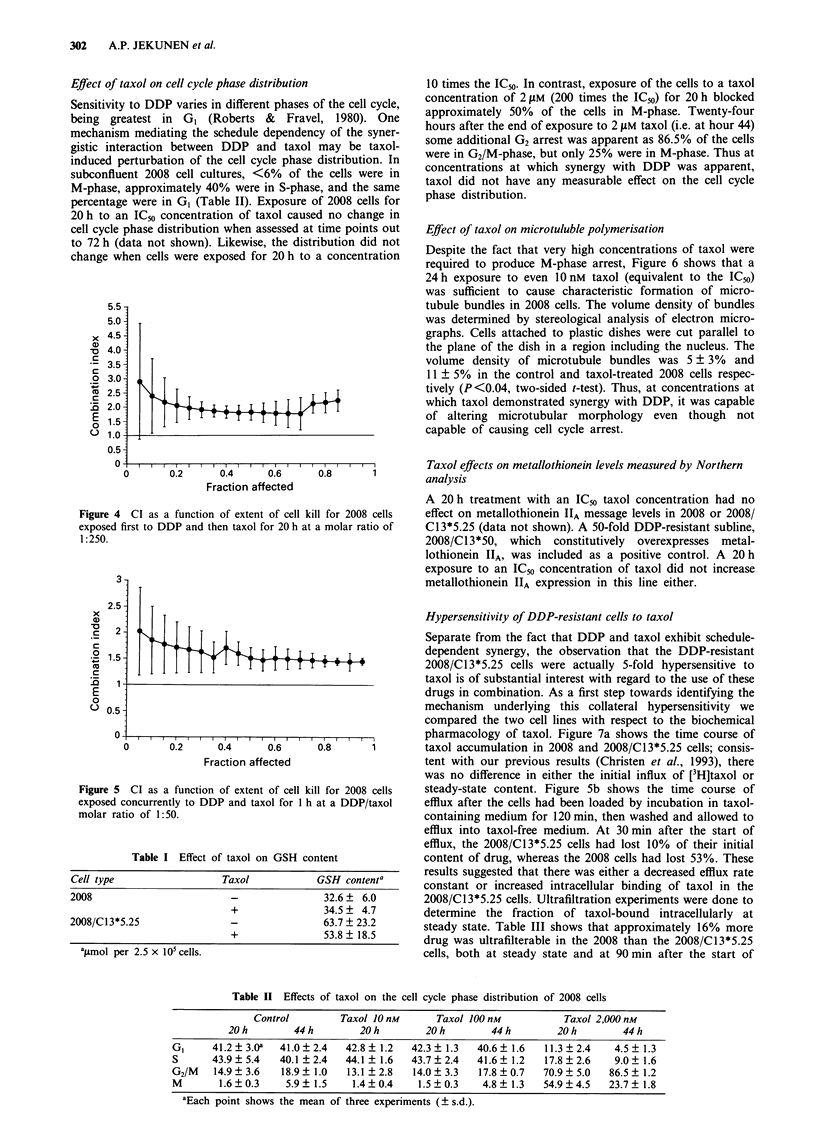

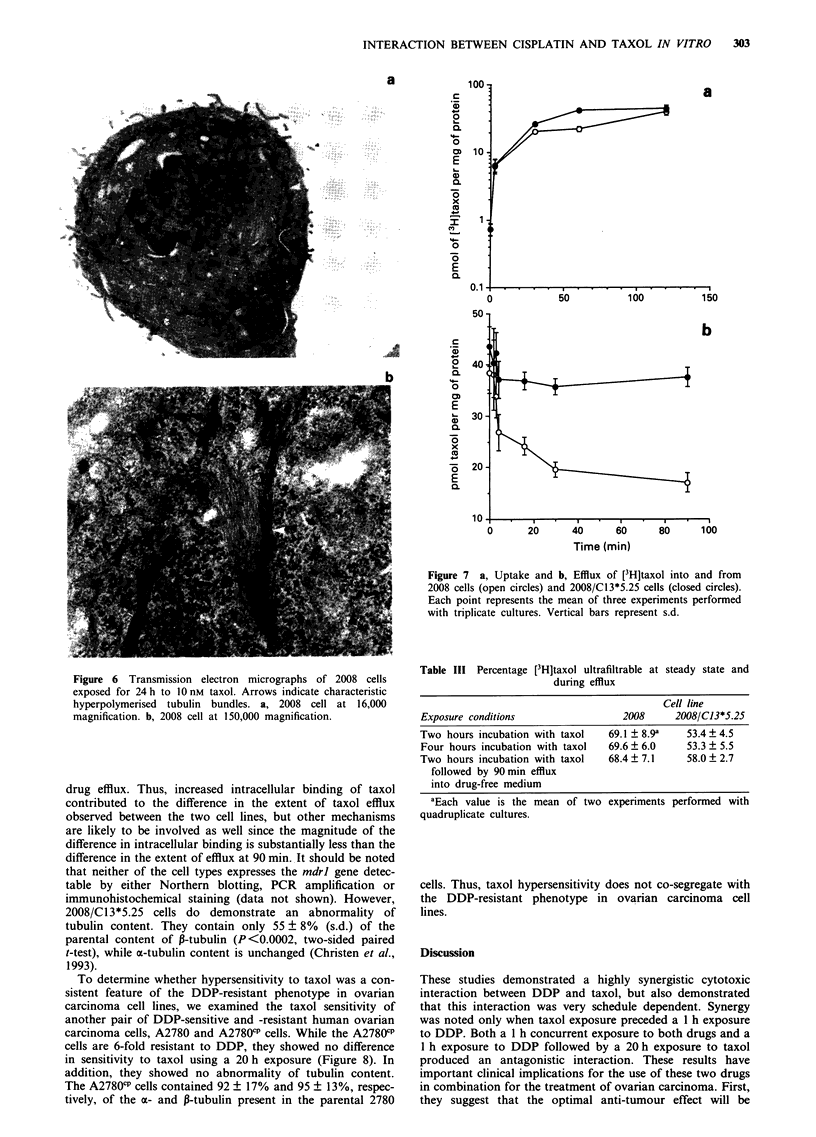

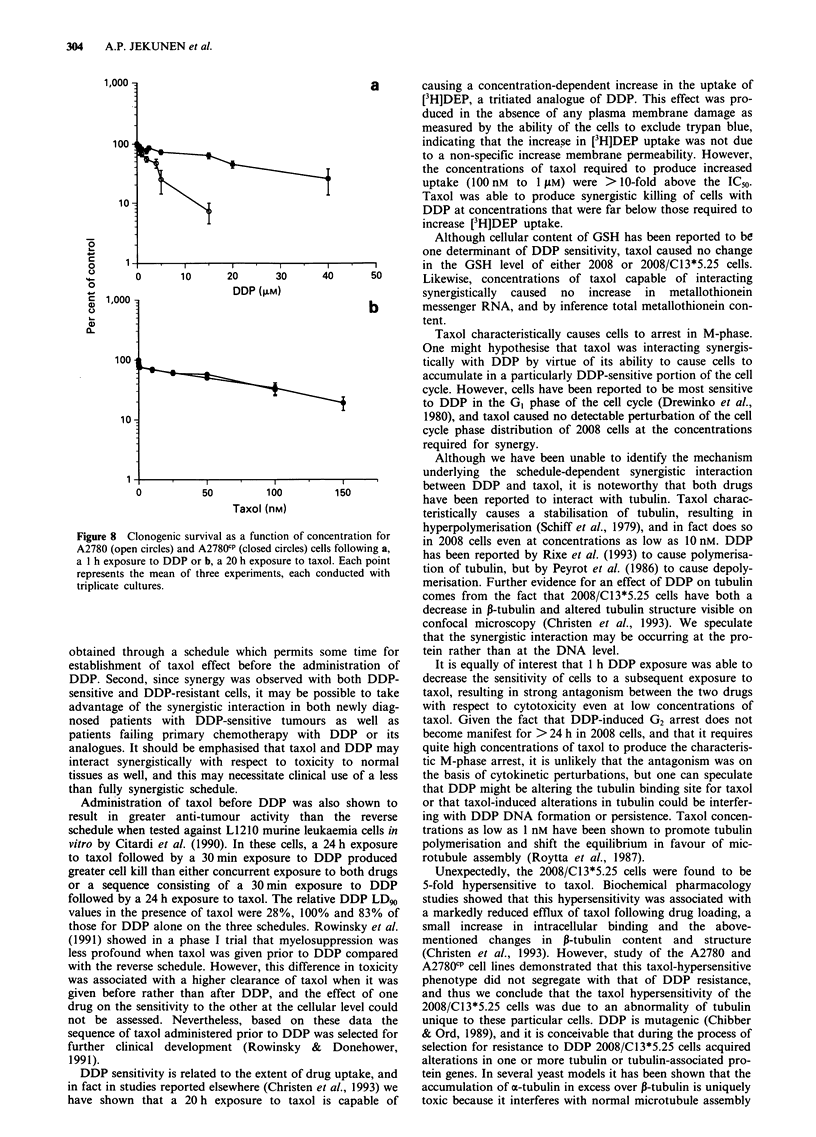

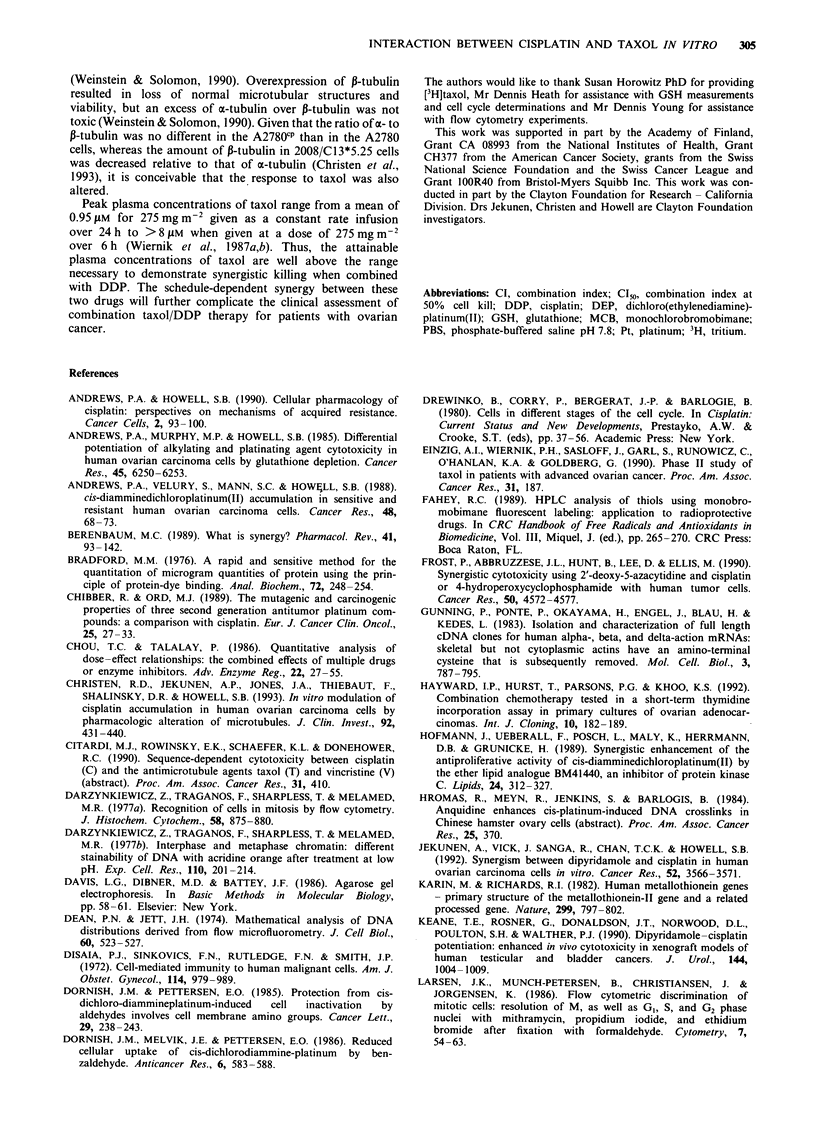

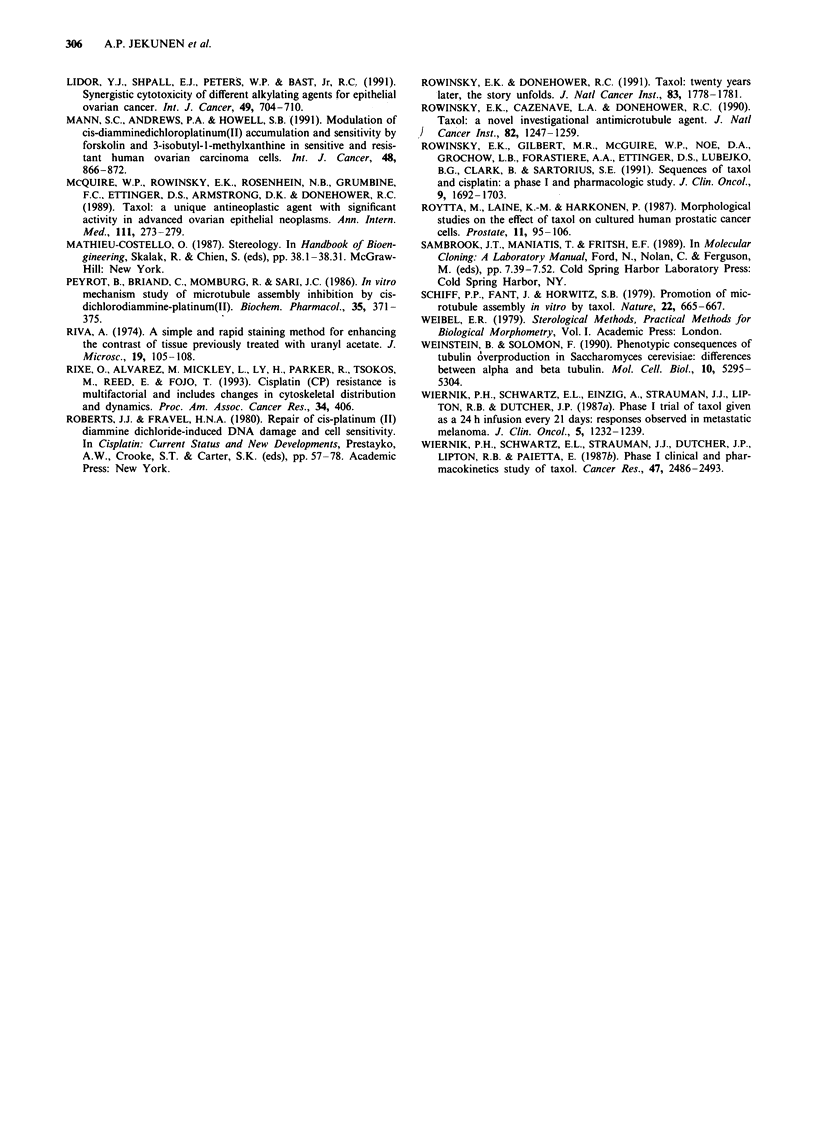


## References

[OCR_00989] Andrews P. A., Murphy M. P., Howell S. B. (1985). Differential potentiation of alkylating and platinating agent cytotoxicity in human ovarian carcinoma cells by glutathione depletion.. Cancer Res.

[OCR_00995] Andrews P. A., Velury S., Mann S. C., Howell S. B. (1988). cis-Diamminedichloroplatinum(II) accumulation in sensitive and resistant human ovarian carcinoma cells.. Cancer Res.

[OCR_01001] Berenbaum M. C. (1989). What is synergy?. Pharmacol Rev.

[OCR_01005] Bradford M. M. (1976). A rapid and sensitive method for the quantitation of microgram quantities of protein utilizing the principle of protein-dye binding.. Anal Biochem.

[OCR_01010] Chibber R., Ord M. J. (1989). The mutagenic and carcinogenic properties of three second generation antitumour platinum compounds: a comparison with cisplatin.. Eur J Cancer Clin Oncol.

[OCR_01016] Chou T. C., Talalay P. (1984). Quantitative analysis of dose-effect relationships: the combined effects of multiple drugs or enzyme inhibitors.. Adv Enzyme Regul.

[OCR_01021] Christen R. D., Jekunen A. P., Jones J. A., Thiebaut F., Shalinsky D. R., Howell S. B. (1993). In vitro modulation of cisplatin accumulation in human ovarian carcinoma cells by pharmacologic alteration of microtubules.. J Clin Invest.

[OCR_01039] Darzynkiewicz Z., Traganos F., Sharpless T., Melamed M. R. (1977). Interphase and metaphase chromatin. Different stainability of DNA with acridine orange after treatment at low pH.. Exp Cell Res.

[OCR_01034] Darzynkiewicz Z., Traganos F., Sharpless T., Melamed M. R. (1977). Recognition of cells in mitosis by flow cytofluormetry.. J Histochem Cytochem.

[OCR_01050] Dean P. N., Jett J. H. (1974). Mathematical analysis of DNA distributions derived from flow microfluorometry.. J Cell Biol.

[OCR_01055] DiSaia P. J., Sinkovics J. G., Rutledge F. N., Smith J. P. (1972). Cell-mediated immunity to human malignant cells. A brief review and further studies with two gynecologic tumors.. Am J Obstet Gynecol.

[OCR_01066] Dornish J. M., Melvik J. E., Pettersen E. O. (1986). Reduced cellular uptake of cis-dichlorodiammine-platinum by benzaldehyde.. Anticancer Res.

[OCR_01060] Dornish J. M., Pettersen E. O. (1985). Protection from cis-dichlorodiammineplatinum-induced cell inactivation by aldehydes involves cell membrane amino groups.. Cancer Lett.

[OCR_01090] Frost P., Abbruzzese J. L., Hunt B., Lee D., Ellis M. (1990). Synergistic cytotoxicity using 2'-deoxy-5-azacytidine and cisplatin or 4-hydroperoxycyclophosphamide with human tumor cells.. Cancer Res.

[OCR_01096] Gunning P., Ponte P., Okayama H., Engel J., Blau H., Kedes L. (1983). Isolation and characterization of full-length cDNA clones for human alpha-, beta-, and gamma-actin mRNAs: skeletal but not cytoplasmic actins have an amino-terminal cysteine that is subsequently removed.. Mol Cell Biol.

[OCR_01104] Hayward I. P., Hurst T., Parsons P. G., Khoo S. K. (1992). Combination chemotherapy tested in a short-term thymidine incorporation assay in primary cultures of ovarian adenocarcinomas.. Int J Cell Cloning.

[OCR_01110] Hofmann J., Ueberall F., Posch L., Maly K., Herrmann D. B., Grunicke H. (1989). Synergistic enhancement of the antiproliferative activity of cis-diamminedichloroplatinum(II) by the ether lipid analogue BM41440, an inhibitor of protein kinase C.. Lipids.

[OCR_01123] Jekunen A., Vick J., Sanga R., Chan T. C., Howell S. B. (1992). Synergism between dipyridamole and cisplatin in human ovarian carcinoma cells in vitro.. Cancer Res.

[OCR_01128] Karin M., Richards R. I. (1982). Human metallothionein genes--primary structure of the metallothionein-II gene and a related processed gene.. Nature.

[OCR_01133] Keane T. E., Rosner G., Donaldson J. T., Norwood D. L., Poulton S. H., Walther P. J. (1990). Dipyridamole-cisplatin potentiation: enhanced in vivo cytotoxicity in xenograft models of human testicular and bladder cancers.. J Urol.

[OCR_01140] Larsen J. K., Munch-Petersen B., Christiansen J., Jørgensen K. (1986). Flow cytometric discrimination of mitotic cells: resolution of M, as well as G1, S, and G2 phase nuclei with mithramycin, propidium iodide, and ethidium bromide after fixation with formaldehyde.. Cytometry.

[OCR_01150] Lidor Y. J., Shpall E. J., Peters W. P., Bast R. C. (1991). Synergistic cytotoxicity of different alkylating agents for epithelial ovarian cancer.. Int J Cancer.

[OCR_01155] Mann S. C., Andrews P. A., Howell S. B. (1991). Modulation of cis-diamminedichloroplatinum(II) accumulation and sensitivity by forskolin and 3-isobutyl-1-methylxanthine in sensitive and resistant human ovarian carcinoma cells.. Int J Cancer.

[OCR_01162] McGuire W. P., Rowinsky E. K., Rosenshein N. B., Grumbine F. C., Ettinger D. S., Armstrong D. K., Donehower R. C. (1989). Taxol: a unique antineoplastic agent with significant activity in advanced ovarian epithelial neoplasms.. Ann Intern Med.

[OCR_01174] Peyrot V., Briand C., Momburg R., Sari J. C. (1986). In vitro mechanism study of microtubule assembly inhibition by cis-dichlorodiammine-platinum(II).. Biochem Pharmacol.

[OCR_01202] Rowinsky E. K., Cazenave L. A., Donehower R. C. (1990). Taxol: a novel investigational antimicrotubule agent.. J Natl Cancer Inst.

[OCR_01198] Rowinsky E. K., Donehower R. C. (1991). Taxol: twenty years later, the story unfolds.. J Natl Cancer Inst.

[OCR_01207] Rowinsky E. K., Gilbert M. R., McGuire W. P., Noe D. A., Grochow L. B., Forastiere A. A., Ettinger D. S., Lubejko B. G., Clark B., Sartorius S. E. (1991). Sequences of taxol and cisplatin: a phase I and pharmacologic study.. J Clin Oncol.

[OCR_01214] Röyttä M., Laine K. M., Härkönen P. (1987). Morphological studies on the effect of taxol on cultured human prostatic cancer cells.. Prostate.

[OCR_01225] Schiff P. B., Fant J., Horwitz S. B. (1979). Promotion of microtubule assembly in vitro by taxol.. Nature.

[OCR_01233] Weinstein B., Solomon F. (1990). Phenotypic consequences of tubulin overproduction in Saccharomyces cerevisiae: differences between alpha-tubulin and beta-tubulin.. Mol Cell Biol.

[OCR_01241] Wiernik P. H., Schwartz E. L., Einzig A., Strauman J. J., Lipton R. B., Dutcher J. P. (1987). Phase I trial of taxol given as a 24-hour infusion every 21 days: responses observed in metastatic melanoma.. J Clin Oncol.

[OCR_01245] Wiernik P. H., Schwartz E. L., Strauman J. J., Dutcher J. P., Lipton R. B., Paietta E. (1987). Phase I clinical and pharmacokinetic study of taxol.. Cancer Res.

